# Supplementary material for: Changes in the Relative Abundance of Two Saccharomyces Species from Oak Forests to Wine Fermentations
Source: Front Microbiol. 2016 Feb 24;7:215. doi: 10.3389/fmicb.2016.00215 (PMC4764737; doi:10.3389/fmicb.2016.00215)
Supplement: Figure S1 — Sampling locations in Slovenia. Forest (green) and vineyard (red) locations are plotted using GPS coordinates of either the start or end of collecting samples. [file Image1.PDF]

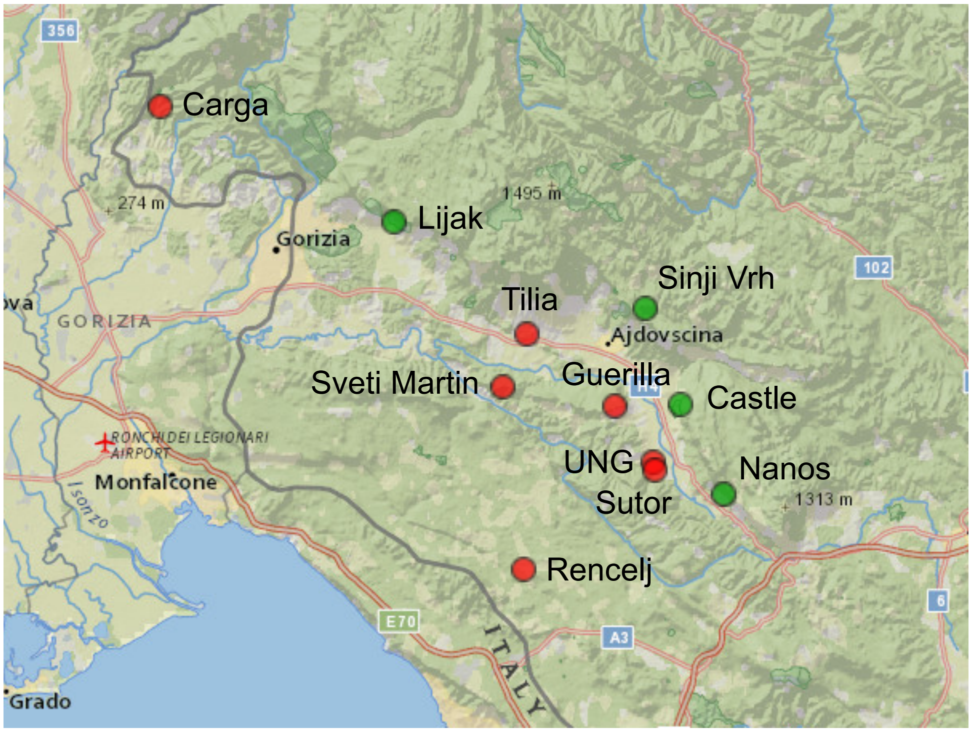

Figure S1. Sampling locations in Slovenia. Forest (green) and vineyard (red) locations are plotted using GPS coordinates of either the start or end of collecting samples.
