# Supplementary figures and images for: Changes in the Relative Abundance of Two Saccharomyces Species from Oak Forests to Wine Fermentations
Source: Front Microbiol. 2016 Feb 24;7:215. doi: 10.3389/fmicb.2016.00215 (PMC4764737; doi:10.3389/fmicb.2016.00215)

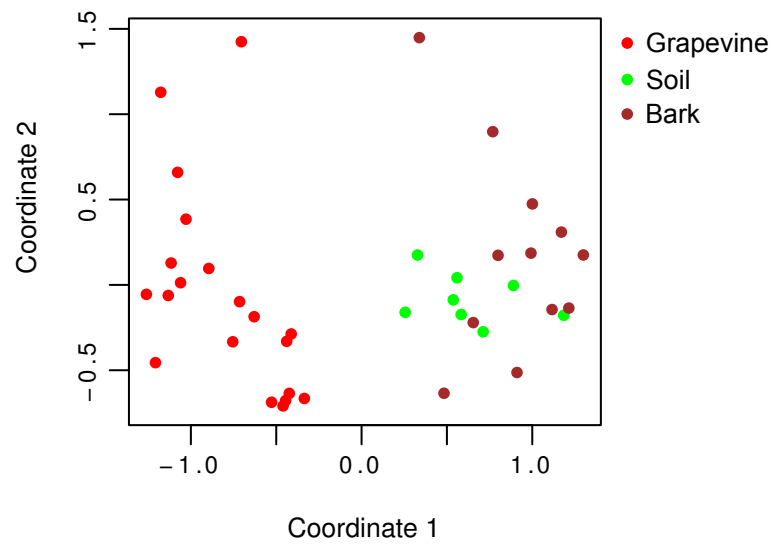

Supplement: Figure S2 — Multidimensional scaling of microbiome samples. First and second coordinates are from non-metric multidimensional scaling using Bray-Curtis dissimilarity. [file Image2.PDF]
