# Supplementary material for: Changes in the Relative Abundance of Two Saccharomyces Species from Oak Forests to Wine Fermentations
Source: Front Microbiol. 2016 Feb 24;7:215. doi: 10.3389/fmicb.2016.00215 (PMC4764737; doi:10.3389/fmicb.2016.00215)
Supplement: Figure S3 — The relative frequency of common species during fermentation. Species with a frequency of 5% or more are shown as a percent of total abundance for Sutor (A, C, E, G) and Burja (B, D, F) fermentations in days. Ferments are Sutor cellar (A), Sutor lab replicates 1-3 (C, E, G) and Burja lab replicates 1-3 (B, D, F). [file Image3.PDF]

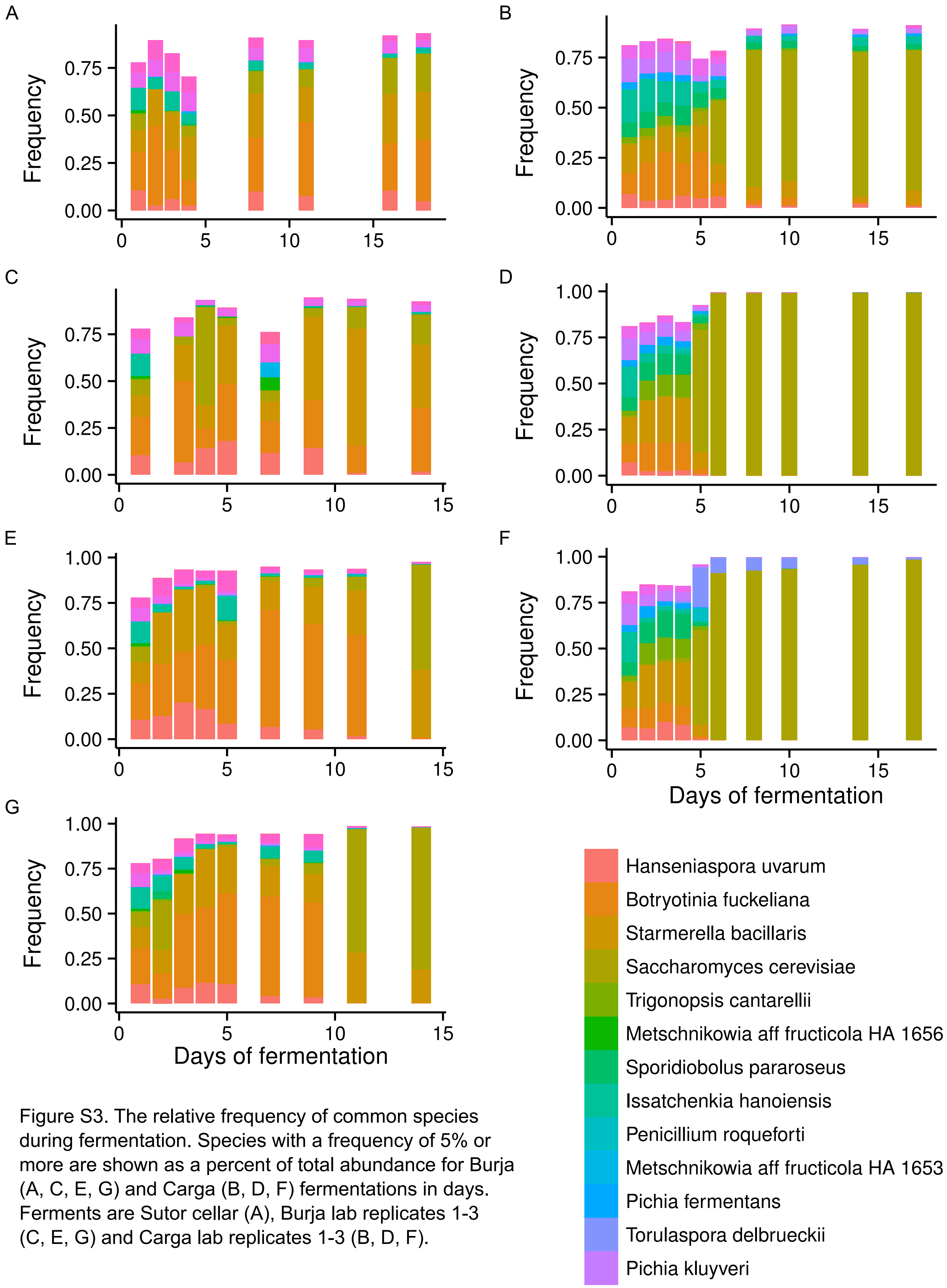

Figure S3. The relative frequency of common species during fermentation. Species with a frequency of 5% or more are shown as a percent of total abundance for Burja (A, C, E, G) and Carga (B, D, F) fermentations in days. Ferments are Sutor cellar (A), Burja lab replicates 1-3 (C, E, G) and Carga lab replicates 1-3 (B, D, F).
