# Supplementary material for: Changes in the Relative Abundance of Two Saccharomyces Species from Oak Forests to Wine Fermentations
Source: Front Microbiol. 2016 Feb 24;7:215. doi: 10.3389/fmicb.2016.00215 (PMC4764737; doi:10.3389/fmicb.2016.00215)
Supplement: Figure S4 — Slovenian and North American strains' resistance to ethanol. Growth rates (AUC) in the presence of ethanol relative to its absence. Black circles and bars represent the mean and its 95% confidence interval. [file Image4.PDF]

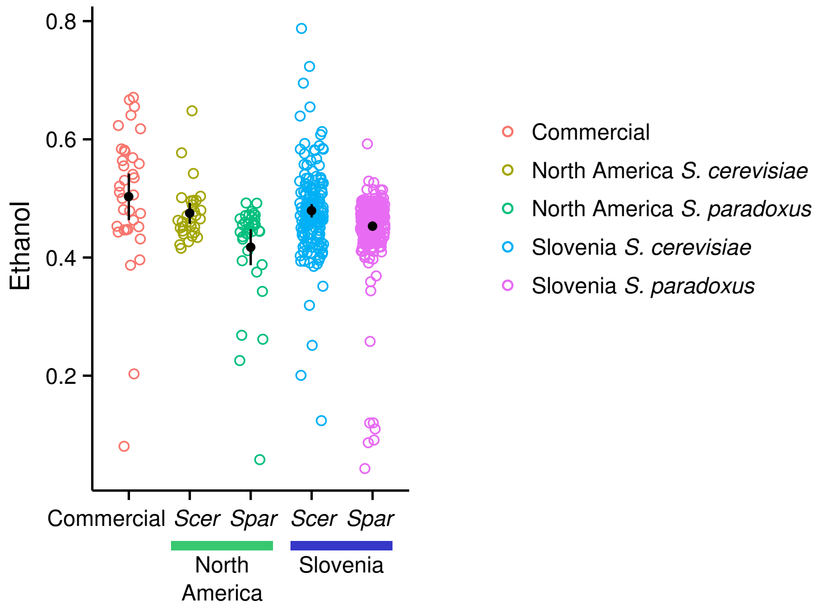

Figure S4. Slovenian and North American strains' resistance to ethanol. Growth rates (AUC) in the presence of ethanol relative to its absence. Black circles and bars represent the mean and its 95% confidence interval.
