# Supplementary material for: Changes in the Relative Abundance of Two Saccharomyces Species from Oak Forests to Wine Fermentations
Source: Front Microbiol. 2016 Feb 24;7:215. doi: 10.3389/fmicb.2016.00215 (PMC4764737; doi:10.3389/fmicb.2016.00215)
Supplement: Figure S5 — Slovenian strains' resistance to sulfites (A), copper (B), tartaric acid (C) and ethanol (D) in relation to location (forest vs vineyard) and source (vine vs oak). [file Image5.PDF]

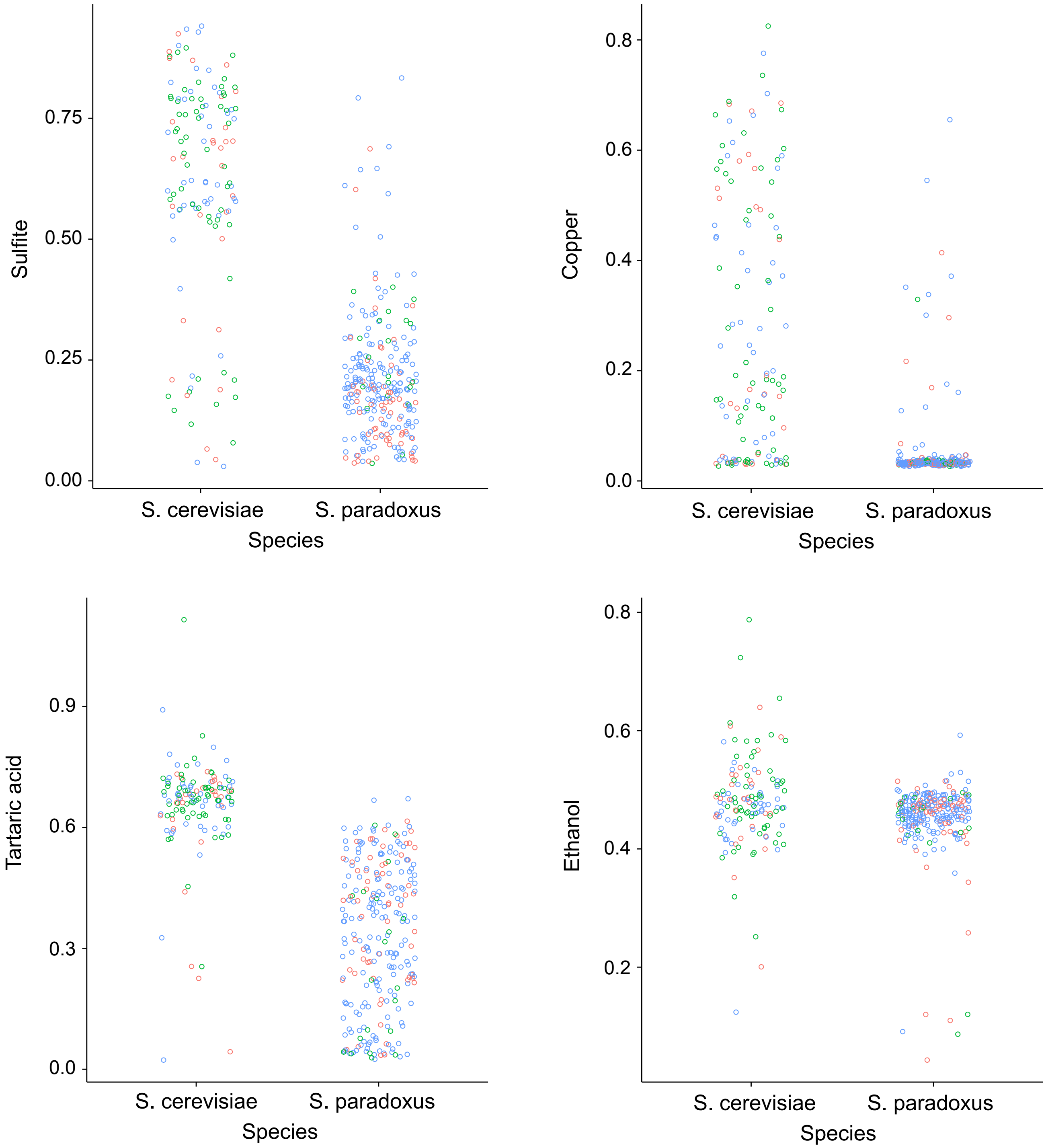

Figure S5. Slovenian strains' resistance to sulfites (A), copper (B), tartaric acid (C) and ethanol (D) in relation to location (forest vs vineyard) and source (vine vs oak).
